# Effects of Modification of Light Parameters on the Production of Cryptophycin, Cyanotoxin with Potent Anticancer Activity, in *Nostoc* sp.

**DOI:** 10.3390/toxins12120809

**Published:** 2020-12-21

**Authors:** Alexandros Polyzois, Diana Kirilovsky, Thi-hanh Dufat, Sylvie Michel

**Affiliations:** 1 Produits Naturels, Analyse et Synthèse, Université de Paris, UMR CNRS 8038 CITCOM, Faculté de Pharmacie de Paris, 75006 Paris, France; thi-hanh.dufat@parisdescartes.fr; 2 Institute for Integrative Biology of the Cell (12BC), CEA, CNRS, Université Paris-Sud, Université Paris-Saclay, 91198 Gif-sur-Yvette, France; diana.kirilovsky@cea.fr

**Keywords:** cyanobacteria, cyanotoxin, light stress, cryptophycin, *Nostoc* sp., abiotic stress

## Abstract

Cryptophycin-1 is a cyanotoxin produced by filamentous cyanobacteria. It has been evaluated as an anticancer agent with great potential. However, its synthesis provides insufficient yield for industrial use. An alternative solution for metabolite efficient production is to stress cyanobacteria by modifying the environmental conditions of the culture (*Nostoc* sp. ATCC 53789). Here, we examined the effects of light photoperiod, wavelength, and intensity. In light photoperiod, photoperiods 24:0 and 16:8 (light:dark) were tested while in wavelength, orange-red light was compared with blue. Medium, high, and very high light intensity experiments were performed to test the effect of light stress. For a 10-day period, growth was measured, metabolite concentration was calculated through HPLC, and the related curves were drawn. The differentiation of light wavelength had a major effect on the culture, as orange-red filter contributed to noticeable increase in both growth and doubled the cyanotoxin concentration in comparison to blue light. Remarkably, constant light provides higher cryptophycin yield, but slightly lower growth rate. Lastly, the microorganism prefers medium light intensities for both growth and metabolite expression. The combination of these optimal conditions would contribute to the further exploitation of cryptophycin.

## 1. Introduction

Cyanobacteria are Gram-negative prokaryotic microorganisms that perform oxygenic photosynthesis. They are a wide group including approximately 150 genera and 2000 species [1]. A large heterogeneity among the genera provides the opportunity for the production of different secondary metabolites with applications in many aspects of people’s lives. These applications vary from biofuels [2,3,4], nutrition [5,6], and biofertilizers [7,8] to cosmeceuticals and pharmaceuticals [9,10]. In that last category, several cyanobacterial toxins were demonstrated to have a possible action as novel anticancer agents [11], as is the case for cryptophycin-1 (Cry) (Figure S1).

Cry is a cyclic depsipeptide that can be isolated from three cyanobacterial strains and from a marine sponge *Dysidea arenaria* in the form of arenastanin A [12]. The three cyanobacteria strains are *Nostoc* sp. MB 5357 [13,14], *Nostoc* sp. GSV 224 [15], and *Nostoc* sp. ASN_M [16]. Now, *Nostoc* sp. MB 5357 is known under the code ATCC 53789 (American Type Culture Collection-ATCC) and is currently the only strain commercially available.

The mechanism of action of Cry is novel. It depletes microtubules in intact cells, leading to cell apoptosis [17]. It is able to interact with the *Vinca* alkaloid binding site of tubulin with high potency and efficacy, but this binding is mechanistically different from previously described compounds with a strong suppression of microtubule dynamics at low concentration [18]. Another major advantage of Cry is that it is a poor substrate for P-glycoprotein (P-gp) mediated transport, linked to multidrug resistance in cancer chemotherapies. Indeed, the overexpression of P-gp, a drug efflux pump, leads to resistance to many natural anti-cancer drugs, such as taxane and *Vinca*-alkaloids, decreasing their efficacy [17,19]. The potent antitumor activity of Cry leads to the development of new pharmaceutical agents [20,21]. That pharmaceutical potential of Cry lead to the need of increasing its production either by culture of *Nostoc* sp. or by chemical synthesis. The chemical synthesis of Cry includes more than 30 steps. In addition, it faces stereoselectivity issues and it has moderate yield (3.5%), which is unprofitable for industrial scale production [22]. So, the search for alternative solutions, such as an increased Cry production from *Nostoc* sp., is necessary. Several culture conditions and medium composition have been tested for *Nostoc* sp. ATCC^®^ 53789™ cultivation and the utilization of BG11 appears to be the best medium composition for optimal metabolite production [13,23,24].

A wide range of environmental conditions for cell culture, including stressful conditions, must be tested in order to find the optimal condition for Cry over-production. Light intensity, photoperiod, and light quality have a big influence on the growth and metabolism of photosynthetic organisms including cyanobacteria. This is the reason why many studies exist about the influence of these parameters on cyanobacteria production of secondary metabolites and growth [25,26,27]. For instance, studies focusing on cyanobacterial growth show that *Nostoc calcicola* has 50% better growth under low light intensities than high intensities [25].

Firstly, in relation to the parameter of the photoperiod, it was observed that the growth rate of cyanobacteria cells increases when the length of the light period increases until it reaches its maximum, which varies from at 16:8 light:dark (L:D) to 22:2 (L:D) depending on the strain [28,29]. It was also demonstrated that the photoperiod influences the production of cyanobacterial toxins. For example, the production of geosmin in *Phormidium* sp. increases when the light portion of photoperiod is increased as well [30].

The light wavelength seems to have an essential role on cyanobacterial performance, as for instance, studies in *Nostoc* sp. and *Synechococcus* sp. strains have shown a positive effect on cell growth under red light and a negative effect under blue light [31,32,33]. Cyanobacteria, like plants, use two pigment complexes, Photosystem II (PSII) and Photosystem I (PSI), to perform photosynthesis. Light is absorbed by the antennae. Red-orange light is absorbed by the major cyanobacteria antenna, the phycobilisome, while blue light is absorbed by the chlorophyll (chl) antennae. Since the PSI has 100 chl molecules and the PSII only has 35, blue light is preferentially absorbed by the PSI. In contrast, the major antenna of the PSII is the phycobilisome and, as a consequence, orange light is preferentially absorbed by the PSII. Red light is absorbed by both chl and phycobilisomes. Thus, illumination by different wavelength causes an imbalance between the photosystems. Blue light was demonstrated to produce an imbalance that is negative for cell growth, since PSII almost does not work [34,35,36]. Regarding the effect of light quality on cyanotoxin production, it was demonstrated that red light has a positive effect on microcystin (MC) production in the cyanobacterium *Microcystis aeruginosa*, while blue light has no effect comparing to white light [37].

Following photoperiod and light wavelength, light intensity is the third most significant light parameter. The effect of light intensity on MC production in several cyanobacteria cells was also studied [27,37,38,39,40,41]. From low to medium light intensity, there is an increase in MC production per cell. Then, the MC production per cell decreases with increasing light intensity [27,37,38,39,40,41]. The rate of this decrease depends on cyanobacterial strain and culture conditions [27,37,38,39,40,41]. This fact explains why in outdoor ponds, the highest amount of microcystin is detected on depths of 1 m and not on the surface. On the surface of the water, the light-intensity is high (around 400 μmol photons m^−2^ s^−1^), while in depths of 1 m, the intensity is much lower and ranges from 20 to 80 μmol photons m^−2^ s^−1^. The growth of cyanobacterial cells is also inhibited by high light intensities that increase the production of dangerous species of oxygen (ROS) and can lead to cell death [25,26,42,43,44,45,46].

In this study, we address the question of how changes in light parameters affect the growth of *Nostoc* sp. ATCC^®^ 53789™ culture and Cry overproduction.

## 2. Results

Under each light condition, the parameters that were examined were cell growth, Cry production per mg of dry biomass, and total Cry production present in 20 mL of culture. The total Cry production depends on the number of cells in the culture and on the Cry production per cell under each light condition. The Cry production per mg of dry biomass gives information about how each light condition influences the synthesis of Cry in each cell.

### 2.1. Effect of Light Intensity

**Figure 1:**
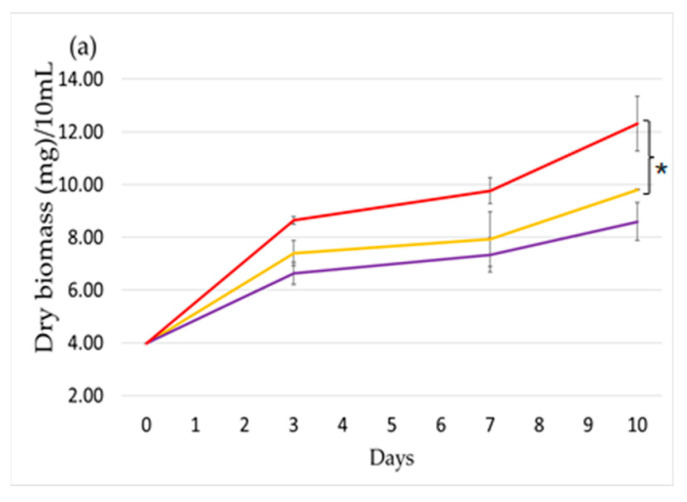

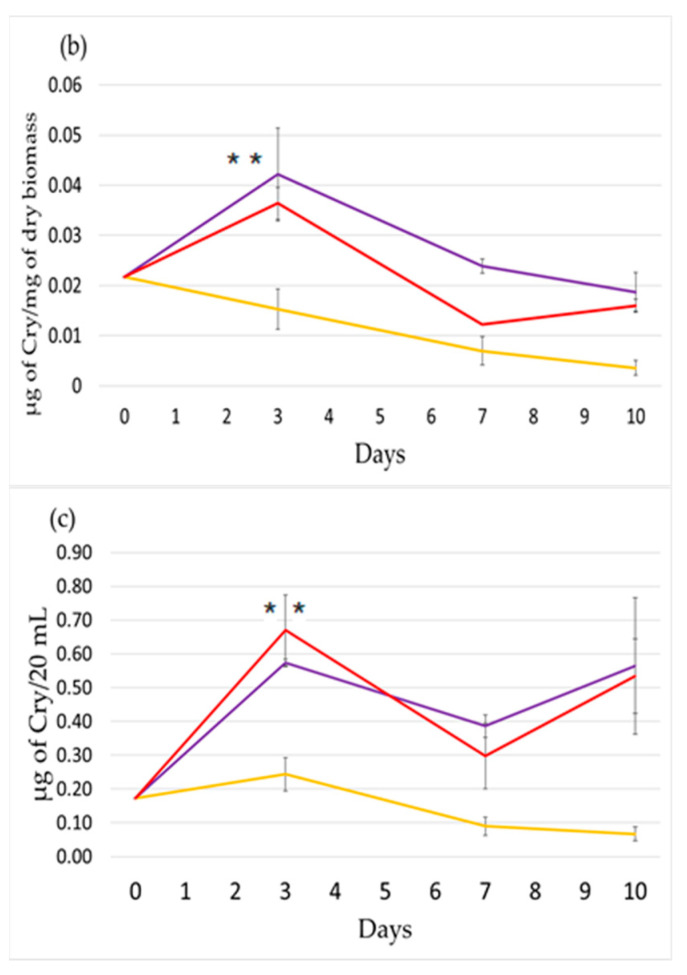
The effect of light intensity on growth and Cry production. (**a**) Growth vs. time, (**b**) Cry production per mg of dry biomass, and (**c**) culture Cry production. 80 μmol photon m^−2^ s^−1^ (red), 120 μmol photon m^−2^ s^−1^ (purple), and 200 μmol photon m^−2^ s^−1^ (yellow). Results are presented as means ± standard deviation (SD) with n = 9 (experiments in biological and technical replicates. A *t*-test has been applied between the maximum of 80 and 200 μmol photon m^−2^ s^−1^ (for t-test and asterisks see the Materials and Methods).

We first studied the effect of light intensity on cell growth and Cry production (Figure 1). The cells were grown in a PSI multi-cultivator using tubes of 5 cm diameter at 25 °C (see details in Materials and Methods). Three light intensities were used: 80 μmol photon m^−2^ s^−1^, 120 μmol photon m^−2^ s^−1^, and 200 μmol photon m^−2^ s^−1^. Under the lowest light intensity, the dry biomass doubled after three days and tripled within 10 days (Figure 1a). Under higher light intensities, the cells grew slower and the biomass doubled only after 8 days of growth (Figure 1a). This shows that the cells are under stress at these light intensities. This was in accordance with microscopic observations showing shorter filaments for cultures subjected to light intensities of 120 and 200 μmol photon m^−2^ s^−1^. The number of shorter filaments increased with the light intensity (Figure S3).

The Cry production per cell was also affected by light intensity (Figure 1b). At point 0, the production of Cry is that of very concentrated cells. Indeed, the experiment began by the dilution of a concentrated *Nostoc* sp. culture (see details in Materials and Methods). Then, during the first three days of the experiment in which the cells are in logarithmic phase of growth, the production of Cry per mg of dry biomass increased under 80 and 120 μmol photon m^−2^ s^−1^. Then, at days 7 and 10, when the culture is still concentrated (and the growth slower than in the first three days), the Cry production decreased, arriving to a level even slightly lower than at point 0. Thus, interestingly, we can see that although 120 μmol photon m^−2^ s^−1^ was already negative for cell growth, it did not decrease Cry production (Figure 1b). In contrast, 200 μmol photon m^−2^ s^−1^ negatively affected both growth and Cry production. As a consequence, in the graph of culture Cry production (Figure 1c), it was observed that Cry concentration in the culture increased during the first three days under 80 and 120 μmol photon m^−2^ s^−1^ light intensity and then decreased while under 200 μmol photon m^−2^ s^−1^. The culture Cry concentration was maintained during the first three days and then decreased. We can conclude that for these experimental conditions, light intensities going from 80 to 120 μmol photon m^−2^ s^−1^ are more convenient for Cry production. More generally, light stress is not suitable for increasing Cry production.

### 2.2. Effect of Light Wavelength

**Figure 2:**
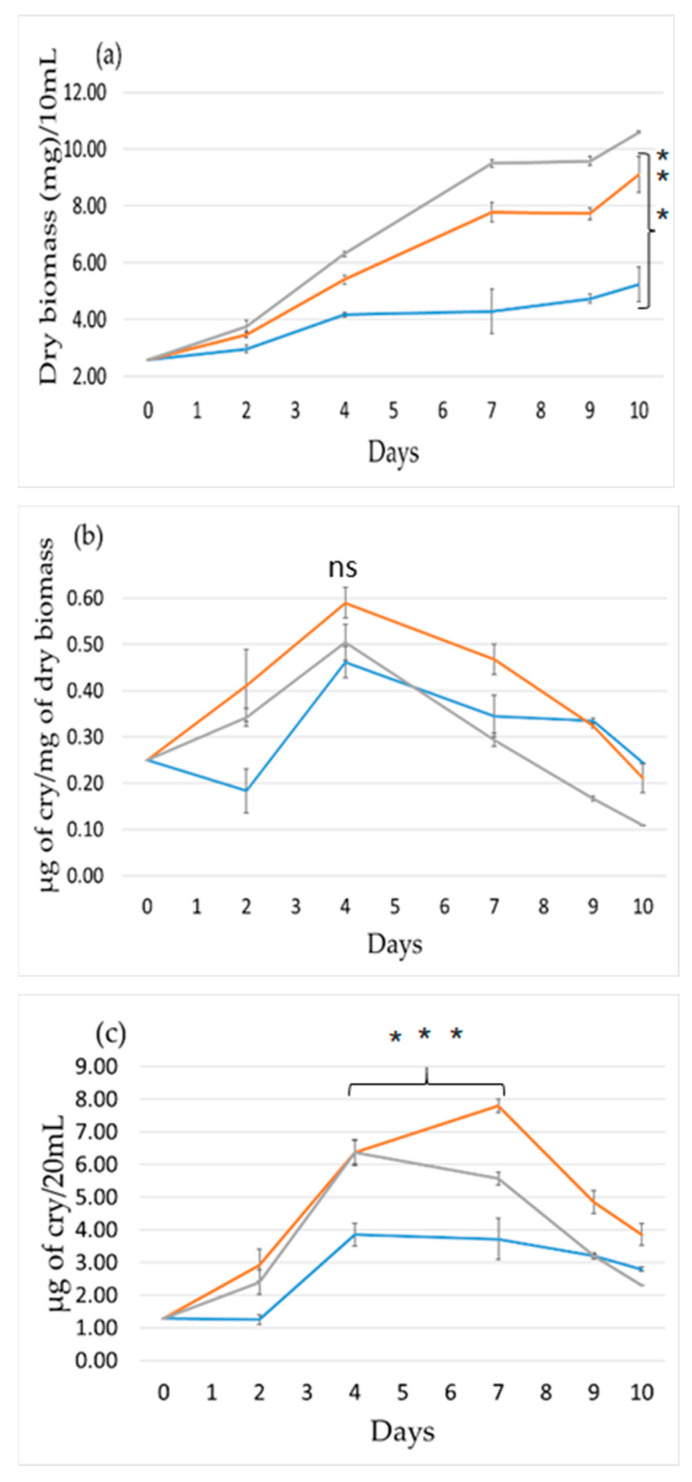
The effect of light wavelength on growth and Cry production. (**a**) Growth vs. time, (**b**) Cry production per mg of dry biomass, and (**c**) culture Cry production. Blue light (blue), orange-red light (orange), and white light (grey). A *t*-test has been applied between the maximum of orange and blue light (for t-test and asterisks see Materials and Methods).

Figure 2 presents the effect of light-wavelength on the culture by comparing orange-red with blue light. For these experiments, *Nostoc* sp. cells were with a light intensity of 45 μmol photon m^−2^ s^−1^ (for more details, see Materials and Methods). Color filters were used to obtain orange-red and blue colors. Due to different culture starting concentration and incubator instrumentation, the growth of cells under these conditions was completely different from that in the light intensity experiment (compare Figure 1 and Figure 2). The curves for white and orange light were close together and tripled after 10 days of growth. In contrast, under blue light, the growth was significantly slower (Figure 2a), as was previously observed in other laboratories [31,32,33], and the biomass only doubled during this 10-day period. Blue light also negatively affected the production of Cry. Specifically, the Cry per cell production under blue light increased only during the period of day 2 to day 4, reaching a maximum of 0.45 μg of Cry/mg of dry biomass. On the other hand, orange and white light increased continuously the Cry per cell production until day 4, when they reached the maximum (doubling of production for white light and 2.5 times higher in orange light) (Figure 2b). Then, the Cry production per cell dropped but faster than under blue light, most probably due to a higher cell concentration. When culture Cry concentration was followed (Figure 2c) under orange-red light, an increase in Cry production was observed for 7 days due to the combination of a higher cell concentration and a higher production of Cry per cell. Instead, under white light, the Cry concentration increased only during the first 4 days. This difference could be related to the larger decrease in Cry production per cell under white light than under orange light. Under blue light, the concentration of Cry increased between days 2 and 4 principally due to the better production of Cry per cell. We conclude that orange light is much better than blue light for Cry production.

### 2.3. Effect Light Photoperiod

**Figure 3:**
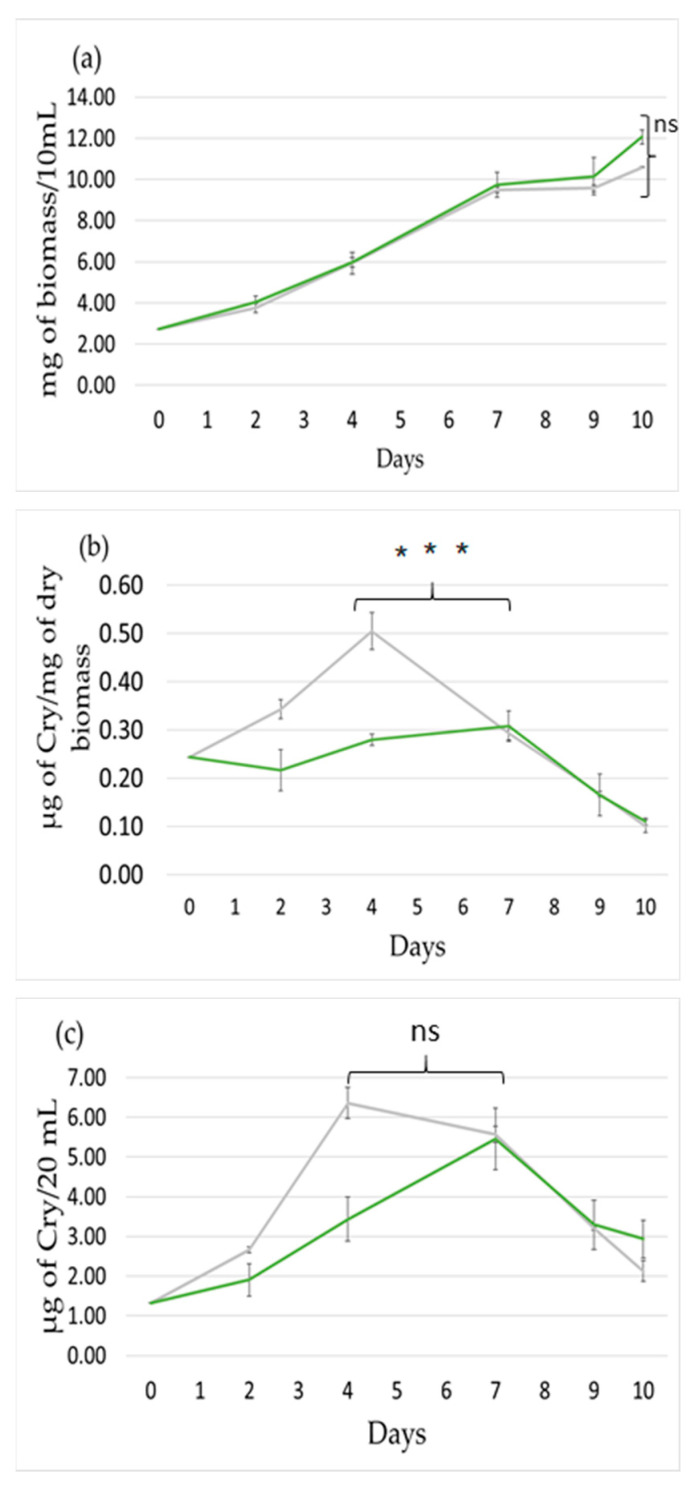
The effect of light photoperiod on growth and Cry production. (**a**) Growth vs. time, (**b**) Cry production per mg of dry biomass, and (**c**) culture Cry production. 24:0 (L:D) (grey) and 16:8 (L:D) (green). A *t*-test has been applied between the maximum of the two photoperiods (for t-test and asterisks see Materials and Methods).

We finally tested whether changes in the photoperiod affect the production of Cry. The *Nostoc* sp. cells for this experiment were also grown in 500 mL Erlenmeyer flasks in a rotary shaker at 25 °C and 45 μmol photon m^−2^ s^−1^ of white light. We compared continuous light (24:0) to a photoperiod of 16 h light and 8 h dark (16:8). The growth rate was similar in both conditions and not a significant difference was observed (Figure 3a). In contrast, the dark period had a very negative effect on Cry production. Specifically, under continuous light, the Cry production per cell increased until the fourth day, when the production doubled as in the previous experiments, and then decreased with increasing cell concentration (Figure 3b). When the photoperiod 16:8 was used, the production of Cry per cell remained low as at point 0 and then, when the cells were concentrated, the production decreased (Figure 3b). As a consequence, the total concentration of Cry in the culture increased faster under continuous light (Figure 3c). We concluded that continuous light is favorable to Cry production, as both per cell and total Cry production are higher in comparison to 16:8 (L:D), until the increased cell concentration which was observed after the fourth day.

## 3. Discussion

Concerning the light intensity study, according to Figure 1, the results showed that medium light intensities (80 μmol photon m^−2^ s^−1^) are suitable for microorganism growth, and that very high intensities are lethal. Very high light intensities (200 μmol photon m^−2^ s^−1^) led to cell death by photoinhibition. The graph is in accordance with the bibliographic data [25,26,42,43,44,45,46], which propose that medium/low light intensities would be optimal. In parallel, the influence of intensity is confirmed regarding the relation between the Cry production and the biomass. The curves of production of Cry at medium-light intensity (120 μmol photon m^−2^ s^−1^) and low intensity (80 μmol photon m^−2^ s^−1^) are very similar, while under very high intensities, the production of Cry is practically stopped. To sum up, in all three graphs on light intensity, in accordance with the bibliographic data [25,26,27,37,38,39,40,41,42,43,44,45,46], a medium light intensity is suitable for growth and metabolite production, and under these conditions, the maximum of that production is on day 3.

Concerning the parameter of light wavelength (Figure 2), orange-red light has a far greater performance both in growth and cyanotoxin production compared to blue light. White light has a similar effect to orange light on growth, but the total production of Cry is higher at day 7 with orange light. Blue spectrum is mostly absorbed only by PSI, so it has significantly lower photosynthetic activity than orange-red light, which can be absorbed by both photosystems [34,35,36]. The lower photosynthetic efficiency of blue light has a negative consequence on both cell growth and Cry production per cell. Consequently, orange-red light is clearly in preference, as it achieves the maximum light efficiency for the growth of the microorganism and the production of the metabolite.

Lastly, as far as the parameter of the photoperiod is concerned, we notice that even if 16:8 (L:D) and constant light have similar effect on growth patterns, the continuous light produces noticeably higher amounts of metabolites. This observation could be explained by two hypotheses about cyanotoxin production. Firstly, the metabolite production might be photo-dependent and light might be a necessary factor for the process. Secondly, under non-light conditions, the microorganism provides the limited produced energy to fulfill its priority of survival and not the secondary metabolite production. Concerning the similar growth patterns, it is indicated that a light period of 16 h is sufficient to store enough energy to maintain cell growth for a non-light period of up to 8 h. In addition, these results suggest that *Nostoc* sp., as with other cyanobacterial strains, shows a preference for the photoperiod of their natural habitat from which they were initially isolated [28]. We observed in all the experiments that the Cry production per cell increased until the day corresponding to a concentration of 7 ± 1 mg dry biomass/10 mL culture, while the slight variation depends on the incubator instrumentation and culture starting concentration. Then, this production was always lower and continued to decrease in the following days. Several causes can contribute to this decrease in production like shear stress, nutrient limitations, and most importantly, the self-shading effect. High cell concentration decreases the light energy reaching each cell due to self-shading; as a consequence, less energy could be used for the production of secondary metabolites that are not essential for cell survival. In the stationary phase, but also in the late exponential phase, changes in the cell metabolism are induced. For example, it is known that in cyanobacteria, the ratio PSII to PSI largely decreased in concentrated cultures. Thus, these metabolism changes could also induce the decrease in Cry production.

To recapitulate, medium intensities of orange-red constant light have been proven to be the optimal light conditions for the amelioration of Cry production by *Nostoc* sp. ATCC^®^ 53789™. Concentrated cultures, which lead to the mentioned self-shade phenomenon, must be avoided to obtain the maximum Cry production per cell. Further research could be done on combining these optimal light conditions with other changes on medium composition, pH and temperature in order to test potential synergic effect. Moreover, scale-up adaptation could be tested with the obtained conditions to demonstrate the possible transition to industrial production.

## 4. Materials and Methods

### 4.1. Cyanobacterial Strain and Culture Conditions 

*Nostoc* sp. ATCC^®^ 53789™ was utilized in liquid suspension cultures and was purchased from ATCC^®^ (American Type Culture Collection, Manassas, VA, USA). The culture medium used was BG-11 [47], to give a mother culture with a concentration of 4.0 mg of dry biomass/10 mL. Each experiment was conducted in technical and biological triplicate (total of nine experiments).

For light-wavelength and photoperiod experiments, the culture was performed in an Erlenmeyer flask of 500 mL, in a Brunswick Innova 4340 Refrigerated Incubator Shaker under constant aeration with a cotton top and under agitation speed of 80 rounds per minute (rpm). The temperature was regulated at 25 ± 1 °C by air conditioning, while illumination with a lamp was performed at an intensity of 45 μmol photon m^−2^ s^−1^. The experiments were conducted in triplicate from each mother cultures. For light-wavelength and photoperiod experiments, each starter inoculum (187.5 mL—4.0 mg of dry biomass/10 mL) was diluted with 112.5 mL of BG11 to give 300 mL of medium with a starting concentration of 2.5 mg of dry biomass/10 mL. For the light wavelength as well as for the light intensity experiment, the photoperiod was 24:0 (L:D).

In order to evaluate the effect of specific wavelength of light, color films (Eurolite, Toronto, ON, Canada) were placed along the external surface of each flask. The transmittance curves of the filters were drawn in a UV-VIS spectrophotometer (Beckman, DU 640, Beckman Coulter Inc., Brea, CA, USA). Based on the curves, the emission spectrum of red filter had a peak at 590–700 nm, and for the blue filter, the peak was at 410–490 nm.

For light intensity experiments, the culture was performed in a multi-cultivator, MC 1000 (Photon System instruments). Each tube had an initial culture volume of 90 mL. The agitation was provided through ambient air bubble column filtered through sterile cotton filter. The temperature was regulated at 25 ± 1 °C using water bath. The difference between the multi-cultivator and the Erlenmeyer flasks led to adapt the initial concentration of the culture. Each tube was inoculated directly from a starter-inoculum at 4.0 mg of dry biomass/10 mL. The light intensity experiments at 80, 120, and 200 μmol photon m^−2^ s^−1^ were conducted from three mother cultures and three technical replicates (nine results).

### 4.2. Growth Measurements

Culture growth was measured through dry biomass calculation. A 0.2 μm porosity nylon filter (Supelco, Nylon 66, 58060-U, 0.20 μm, Supelco, Inc., Merck France, Molsheim, France) was heated at 105 °C for 2 h and weighed. Then, 10 mL of homogenized sample were filtered, and 10 mL more of H_2_O were added right after to wash away the remaining salts. Lastly, the filter was heated again at 105 °C for 4 h and weighed. The dry biomass was calculated as the mean of three samples. Every sampling day, the culture was examined through microscopic observations with optical microscope at ×10 and ×40 magnification in order to verify the absence of contaminants and to check the physiology of the filaments and their possible fragmentations.

### 4.3. Cry Preliminary Quantification

Sample preparation is carried out from 20.0 mL of culture medium taken every day in the morning, under sterile conditions. The sample was centrifuged at 2000 rpm for 25 min (BR4i—Jouan centrifuge) at 25 °C. Then, the supernatant was discarded, and the lower solid phase was transferred quantitatively in a PRECELLYS^®^ tube and recentrifuged at 10,000 rpm for 10 min (1–14—Sigma centrifuge) at 25 °C. The upper phase was discarded, and the residual solid phase was suspended with 500 μL of acetonitrile (MeCN). To extract Cry from the biomass cells, cellular walls were disrupted using 0.5 g of beads (1.0–1.4 mm diameter) in each tube placed in the PRECELLYS^®^ 24 Tissue homogenizer (Bertin instruments) at 5000 rpm, for 3 rounds of 20 sec with a 5 sec stop after each round. The tube was then centrifuged at 10,000 rpm for 1 min (Figure S4) (1–14—Sigma centrifuge). The final upper phase was subjected to High Performance Liquid Chromatography (HPLC) analysis using a reversed phase column (Zorbax Eclipse Plus-C18, 4.6 × 150 mm i.d., 5 μm, precolumn Zorbax Eclipse Plus-C18, 4.6 × 12.5 mm i.d., 5 μm). We applied gradient mode with H_2_O/MeCN (MeCN 50% at 0 min,70% at 60 min, 100% 65–75 min), a flow rate of 1 mL/min, a UV detection wavelength at 234 nm, and an injection volume of 20 μL. Under these conditions, the Cry retention time was 22.85 ± 0.03 min. The Cry quantification was calculated from the equation of a standard curve obtained from 9 concentrations of Cry solution, from 0.10 to 27.83 μg/mL. Internal laboratory Cry reference was used, the purity of which was previously established (>95%) (Figure S2). The calculation is based on the following formula: y = 1.735 × x, where y is the concentration of Cry (μg/0.5 mL), and x is the area under the curve (AUC) of Cry peak measured at 235 nm.

### 4.4. Statistical Analysis 

Experimental values were presented as means ± standard deviation. The related graphs of productivity and growth were drawn using Microsoft Excel software. A *t*-test has been applied on the three light tests, where ns for *p* > 0.05, * for *p* ≤ 0.05, ** for *p* ≤ 0.01, and *** for *p* ≤ 0.001.

## Supplementary Materials

The following are available online at https://www.mdpi.com/2072-6651/12/12/809/s1, Figure S1: Structure of cryptophycin 1, Figure S2: Cryptophycin-1 standard, Figure S3: Microscopic observation of *Nostoc* sp. ATCC 53789, Figure S4: Comparative chromatogram of cryptophycin-1 and crude extract.
