# Supplementary material for: Effects of Modification of Light Parameters on the Production of Cryptophycin, Cyanotoxin with Potent Anticancer Activity, in Nostoc sp
Source: Toxins (Basel). 2020 Dec 21;12(12):809. doi: 10.3390/toxins12120809 (PMC7766261; doi:10.3390/toxins12120809)
Supplement: Supplementary file 1 [file toxins-12-00809-s001.pdf]

Toxins **2020**, *12*, 809; doi:10.3390/toxins12120809

Supplementary Materials: Effects of Modification of Light Parameters on the Production of Cryptophycin, Cyanotoxin with Potent Anticancer Activity, in *Nostoc* Sp.

Alexandros Polyzois, Diana Kirilovsky, Thi-hanh Dufat and Sylvie Michel

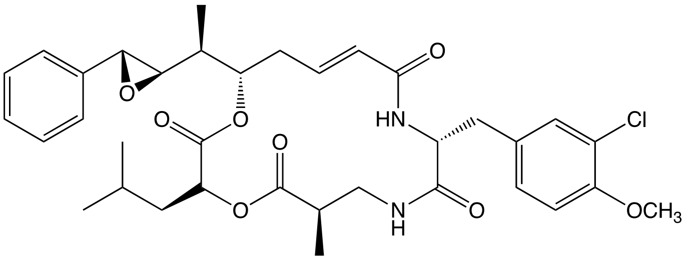

Figure S1. Structure of cryptophycin 1.

HRMS:
$C_{35}H_{44}ClN_2O_8$ $[M+H]^+$:
Exact mass m/z: 655.277709 measured, m/z: 0.655.2760070 calc., error -0,6 ppm
HPLC-DAD profiling

Thermo Scientific/ Ultimate 3000 / software Chromeleon 6.8.<br>Column: Zorbax Eclipse Plus C18, 4.6 i.d. x 150 mm, 5 µm<br>Precolumn: Zorbax Eclipse Plus C18, 4,6 i.d. x 12,5 mm, 5 µm<br>Solvent gradient CH_3CN- H_2O, 0 – 60 min: CH_3CN: 50-> 70, flow: 1 mL/min	
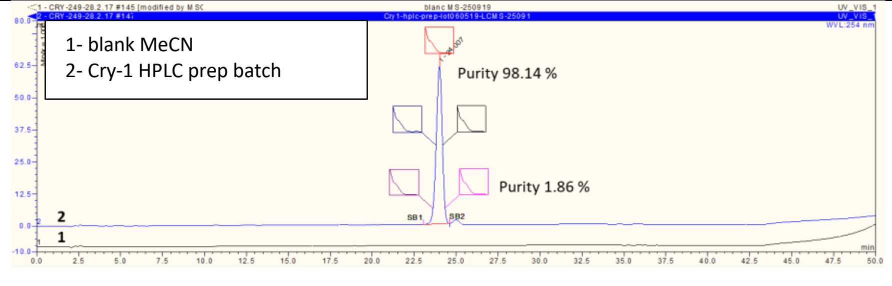	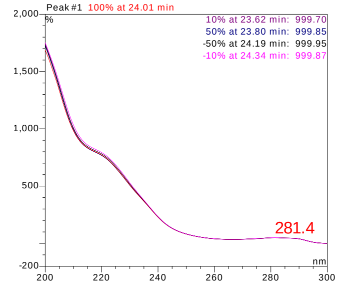

^{1}H NMR spectra (5 mg/0,7 mL $CDCl_3$ – 600 MHz)

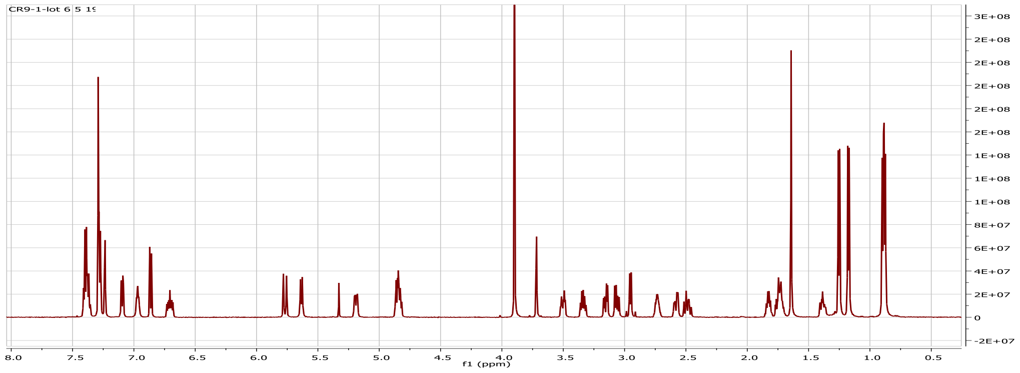

^{13}C NMR spectra (5 mg/0,7 mL $CDCl_3$ – 400 MHz)

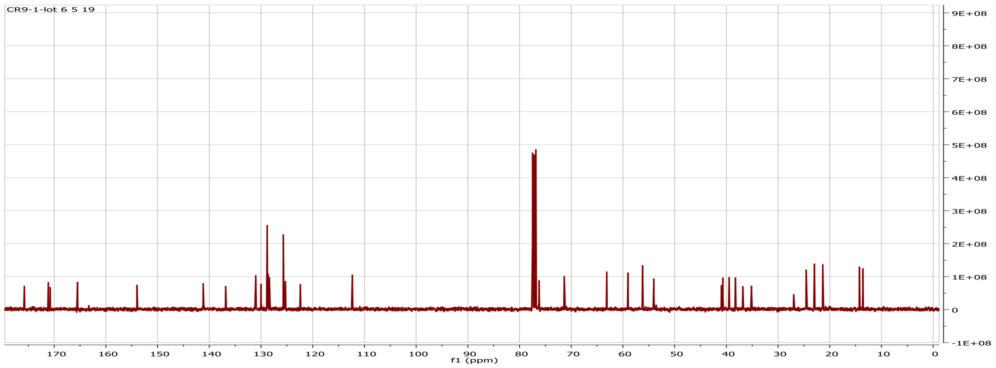

Figure S2. Cryptophycin-1 standard.

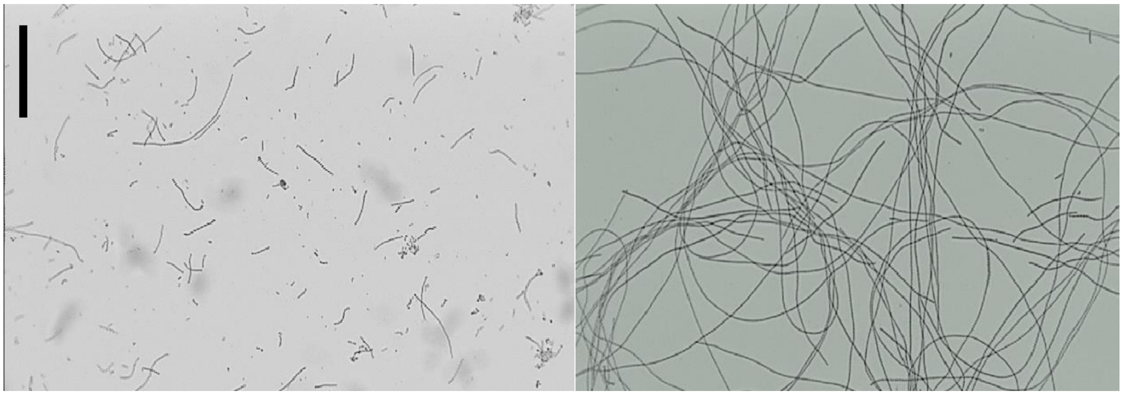

.1Fragments of filaments of Nostoc sp. ATCC 53789 after photoinhibition at 200 (left pannel), filaments of Nostoc sp. ATCC 53789 at 80. The line is 1.9 mm. (microscopic observation using Motic Finite optical Microscope B1-220E-SP)

Figure S3. Microscopic observation of Nostoc sp. ATCC 53789.

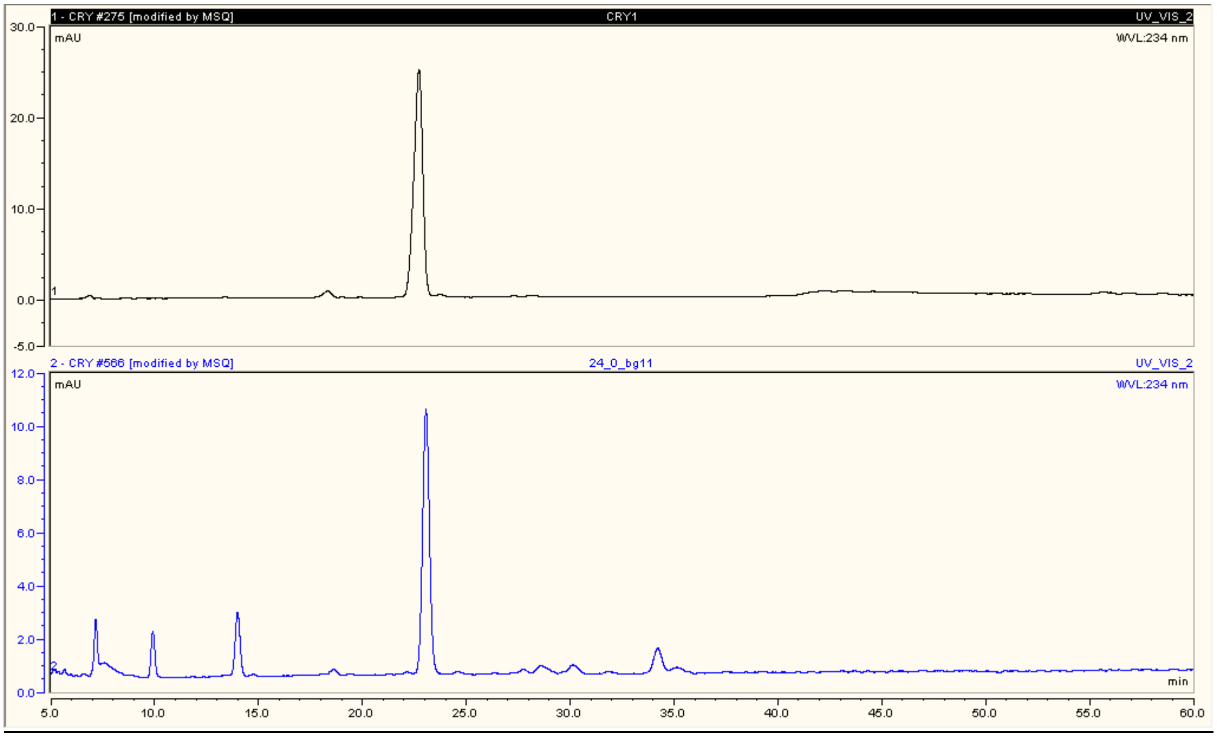

Comprative chromatogram of cryptophycin -1 standard (upper pannel) and crude extract of Nostoc sp. ATCC 53789. The crude extract is from the experiment of photoperiod, from the set of 24:0 (L:D). The chromatogrm is focused on 5.0-60.0 min to present the part related to cryptophycin. (For conditions see materials and methods)

Figure S4. Comparative chromatogram of cryptophycin-1 and crude extract.
